# Clinical and translational research workforce education survey identifies needs of faculty and staff

**DOI:** 10.1017/cts.2021.875

**Published:** 2021-11-10

**Authors:** Tammy L. Loucks, Jillian Harvey, Diana Lee-Chavarria, Rechelle Paranal, Kathleen A. Lenert, Heather S. Bonilha, Carol Feghali-Bostwick

**Affiliations:** 1 South Carolina Clinical and Translational Research Institute, Medical University of South Carolina, Charleston, South Carolina, USA; 2 Academic Affairs Faculty, Medical University of South Carolina, Charleston, South Carolina, USA; 3 Department of Health Care Leadership and Management, College of Health Professions, Medical University of South Carolina, Charleston, South Carolina, USA; 4 Department of Rehabilitation Science, College of Health Professions, Medical University of South Carolina, Charleston, South Carolina, USA; 5 Division of Rheumatology and Immunology, Department of Medicine, College of Medicine, Medical University of South Carolina, Charleston, South Carolina, USA

**Keywords:** Professional development, research training, learning preferences, translational research workforce

## Abstract

Developing the translational research workforce is a goal established by the National Center for Advancing Translational Science for its network of Clinical and Translational Science Award Program hubs. We surveyed faculty and research staff at our institution about their needs and preferences, utilization of existing trainings, and barriers and facilitators to research training. A total of 545 (21.9%) faculty and staff responded to the survey and rated grant development, research project development, and professional development among their top areas for further training. Faculty prioritized statistical methods and dissemination and implementation, while staff prioritized research compliance and research administration. Faculty (73.9%; n = 119) and staff (87.3%; n = 165) reported that additional training would give them more confidence in completing their job responsibilities. Time and lack of awareness were the most common barriers to training. Our results indicate the value of training across a range of topics with unique needs for faculty and staff. This pre-COVID survey identified time, awareness, and access to training opportunities as key barriers for faculty and staff. The shift to remote work spurred by the pandemic has further heightened the need for effective and readily accessible online trainings to enable continuous development of the clinical and translational research workforce.

## Introduction

Training and cultivating the translational science workforce are one of the five major goals established by the National Center for Advancing Translational Sciences for its network of Clinical and Translational Science Award (CTSA) Program hubs [1]. As the primary academic health science center for the state of South Carolina and member of the CTSA network, our institution is committed to the development of a clinical and translational research workforce equipped to lead and enable research that effectively addresses the health needs of our population. The Translational Workforce Development (TWD) program for our CTSA hub seeks to provide comprehensive and accessible training for all individuals engaged in clinical and translational research and works collaboratively across the institution to identify new training needs and develop synergistic training opportunities that support the development and retention of a diverse research workforce.

Providing training opportunities is the primary method used to develop a clinical and translational research workforce and continue to grow their knowledge and skills. At our hub, these opportunities vary from 1-h Lunch & Learns to multiweek courses to multiyear career development and training programs. Several different entities at our institution, including but not limited to the CTSA, the Advancement, Recruitment, and Retention of Women Program [2], the Mentor Leadership Council [3], and research support offices, offer training and professional development for the research workforce. The topics and intended audiences of these opportunities vary widely, and, while many are well-attended, we frequently hear from faculty and staff that they are not aware of the offerings. In addition, the expansion of the clinical enterprise and hectic work schedules may leave some faculty and staff unable to attend in-person training sessions at the main university campus. Given that training of faculty and staff is critical for meeting the evolving demands of a diverse workforce, it is important that relevant learning opportunities are accessible.

To meet our goal of providing comprehensive and accessible learning opportunities for all individuals engaged in clinical and translational research, we seek to offer courses and trainings that meet the varied educational needs of our workforce. Our charge is to offer training in areas that ensure our workforce is prepared to meet the challenges of, and contribute to, the evolving and increasingly complex field of clinical and translational research. As part of this goal, we previously compiled a list of all research-related trainings across our institution to help build a central portal for training opportunities and to analyze redundancy and gaps. To further guide ongoing development of TWD program offerings, we surveyed faculty and research staff at our institution about their research training needs and preferences, utilization of existing trainings, and barriers and facilitators to participating in research training.

## Methods

A survey was developed to assess the training needs and preferences of the institution’s research workforce as part of program improvement and not considered human subjects research. Survey questions were developed through an iterative process [4] prior to distribution by a multidisciplinary team of research investigators, education and training specialists, evaluators, and CTSA program managers representing Research Coordination and Management, Regulatory Knowledge and Support, and Workforce Development functions. Questions were assessed for face validity by team members with expertise in research workforce training and education. Next, two experts in survey methods and program evaluation assessed the survey for methodological errors and adherence to survey best practices [5]. Finally, the survey was piloted by the team for survey functionality, content, clarity, ease of understanding, usefulness, and comprehensiveness.

REDCap [6] was used to administer anonymously, the survey that consisted of 18 questions including one question allowing recipients to opt out if they were not involved in the direct conduct, support, oversight, or administration of research (see Supplemental Material). The survey was emailed in October of 2019 to all faculty (n = 1600) and research staff (n = 893) at the institution. Only staff in research-focused job classifications, which included staff involved in the direct conduct as well as the administration, oversight, and support of research, were selected to receive the survey. Staff in nonresearch job classifications were excluded. The survey was available for 2 weeks, and faculty and staff received one reminder during this interval.

Position (faculty or staff), rank (faculty), job title (staff), research area (basic, clinical, translational, or other), department affiliation, time in current position, time conducting research, as well as information on race, ethnicity, and gender, were collected. Respondents were asked to rate the perceived value of 11 topic areas and four learning initiatives as very valuable, somewhat valuable, or not valuable. Topic areas were selected based on evaluations and feedback from current trainings, areas of CTSA focus, and ECPTRQ competencies [7]. Respondents were able to provide open-ended feedback to identify specific examples of trainings for topics they identified as very valuable, suggest other areas not listed that would be valuable training topics or important for new research orientation, and explain barriers to participation in trainings. Participation in institutional research trainings in the previous 2 years, type and sponsoring program for the trainings were also captured. Respondents were asked to rate the effectiveness of four training methods that included online, blended (a mix of online and in-person), and live formats, and a resource library. They were also asked to indicate the likelihood of attending an online, blended, or live training. Finally, respondents were queried as to whether they knew where to look for training opportunities and their preferred method of receiving information about research learning opportunities.

Respondent demographic characteristics are reported as means and standard deviations for continuous variables and counts and proportions for categorical variables. Descriptive statistics for ratings of topics, learning initiatives, effectiveness of training approaches, and likelihood of attending a training are presented separately for faculty and staff.

The open-ended survey questions were analyzed using a general inductive approach for analysis of qualitative evaluation data, which provides a systematic approach to summarizing data into actionable themes [8]. Two researchers, trained in qualitative methods, read the qualitative data multiple times to identify relevant categories related to the workforce training needs and barriers through an iterative process. Initial categories were developed through independent reading of the survey data. Six high-level categories aligned with the evaluation aims (barriers, new training opportunities, orientation, participation, and value). Specific subcategories were developed through multiple readings of the data. The final set of codes was applied to all open-ended question responses. NVivo software was utilized to facilitate and organize analysis. The categories were then compared and contrasted across both staff and faculty respondent types to identify emerging themes.

## Results

### Quantitative Results

**Fig. 1.**
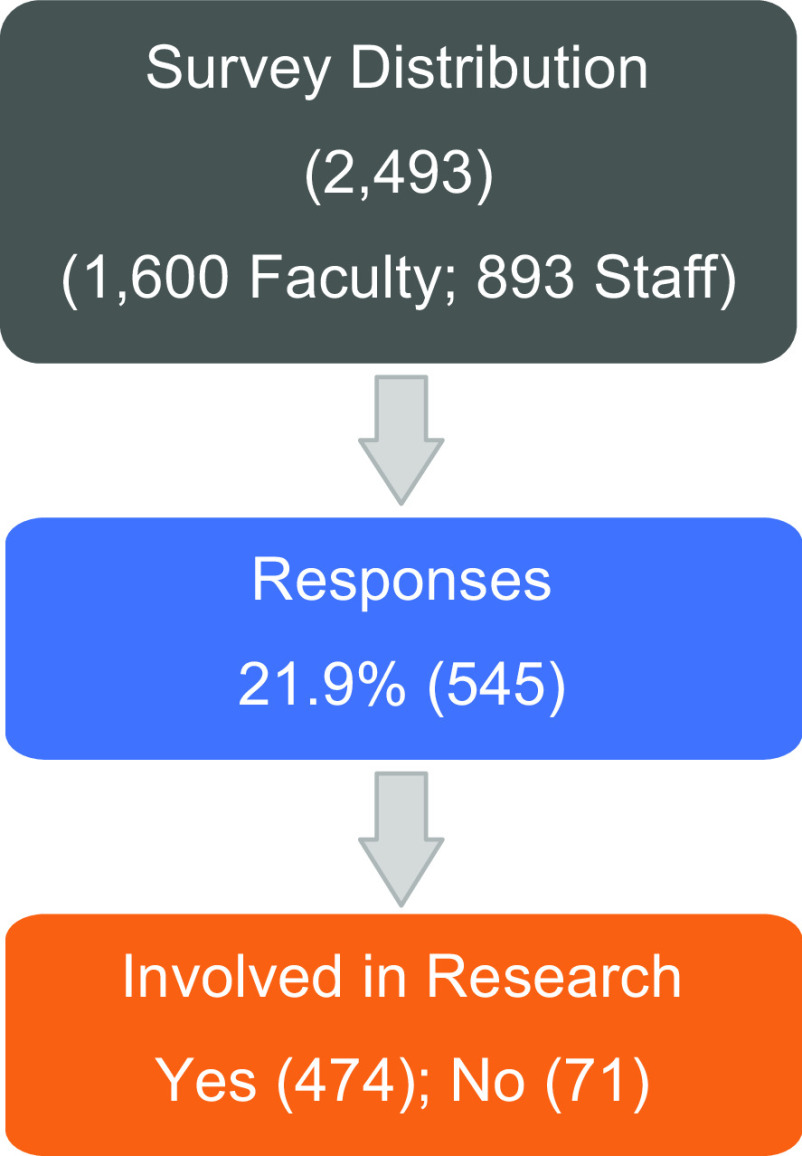
Diagram of survey participants.

The survey was emailed to 2493 individuals and 545 (21.9%) responded (Fig. 1). Eighty-seven percent (n = 474) of those responding indicated that they were involved in research. Among those indicating that they were involved in research, 41.6% (n = 197) held faculty positions and 52.1% (n = 247) were in a staff role. Respondents who did not identify their role (6.3%, n = 30) and those indicating that they were not involved in research (13%, n = 71) were excluded from the remaining analyses. Characteristics of the respondents included in the analyses are presented in Table 1.

**Table 1.** Descriptive information

Demographic	Faculty	Staff	All
Gender (n = 285)			
Female	64.2%	75.2%	70.5%
Male	31.7%	17.6%	23.5%
Not reported	4.2%	7.3%	6.0%
Race (n = 283)			
Caucasian	79.0%	75.0%	76.7%
African American	4.2%	7.3%	6.0%
Other races	9.2%	10.4%	9.9%
Not reported	7.6%	7.3%	7.4%
Time in position at MUSC, years (n = 337)	5.8 (5.6)	5.0 (5.5)	5.4 (5.6)
Time in research, years (n = 396)	14.6 (9.4)	8.7 (7.8)	11.4 (9.0)
Type of research (n = 444)			
Basic	25.4%	23.1%	24.1%
Clinical	48.7%	49.8%	49.3%
Translational	21.3%	22.3%	21.8%
Other (education/administration)	4.6%	4.9%	4.7%

%(n) or mean (standard deviation). MUSC, Medical University of South Carolina.

**Fig. 2.**
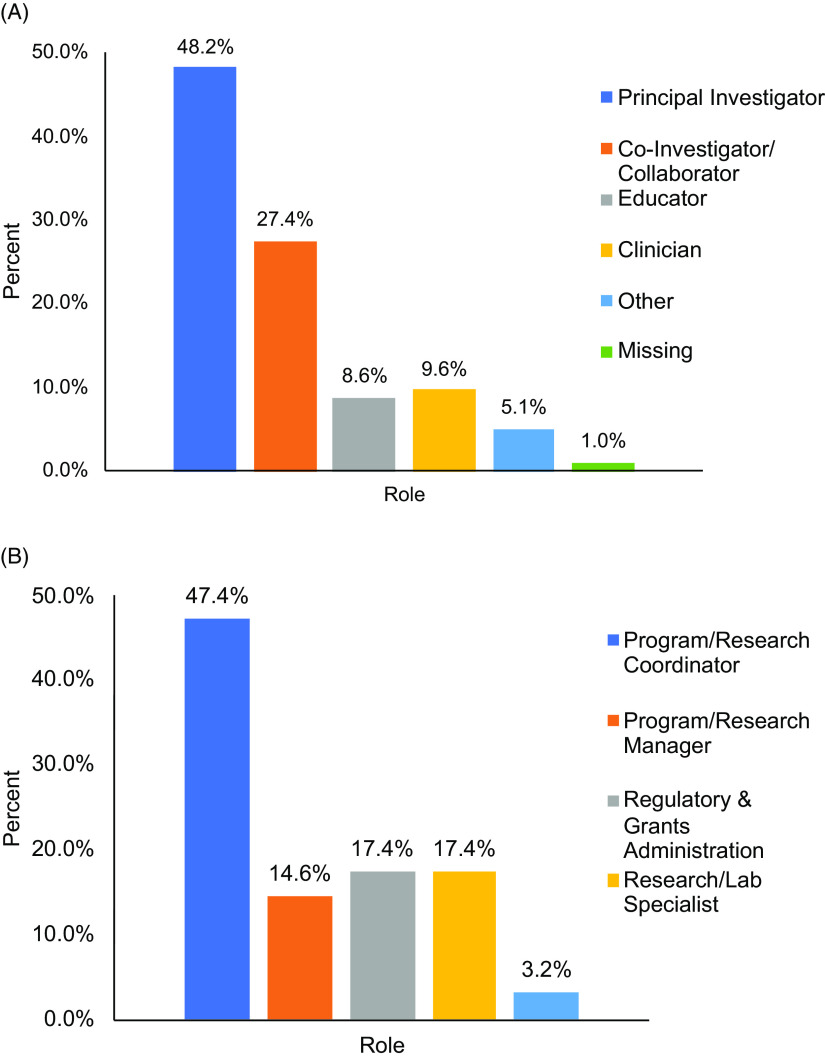
Distribution of faculty (A) and staff (B) by role in research.

Faculty were primarily affiliated with the College of Medicine (COM) 71.6% (n = 141), which is the largest of the six colleges at our institution. Faculty at the level of professor (22.8%), associate professor (28.9%), assistant professor (38.1%), and instructor (5.6%) were represented among the respondents. Nearly half of faculty (48.2%) identified themselves as primary investigators of a research (n = 76) or a career development award (n = 19) (Fig. 2A). Staff affiliations followed a similar pattern with 76.1% (n = 188) reporting an association with the COM. As shown in Fig. 2B, staff represented diverse research roles including program and project coordination, program management, regulatory and grants administration, laboratory specialists, and staff scientists.

All respondents were asked to rate the perceived value of 11 topic areas. Topics are listed in the order of highest perceived value as identified by faculty and staff in Table 2.

**Table 2.** Proportion of faculty and staff who rated educational topics as very valuable

Topic area	Faculty (178)	Topic area	Staff (213)
Grants development	72.5%	Research compliance	78.4%
Statistical methods	68.5%	Professional development	75.1%
Research project development	68.5%	Research administration	65.7%
Professional development	65.7%	Research project development	63.8%
Dissemination and implementation	60.1%	Grants development	59.0%
Scientific communication	48.0%	Dissemination and implementation	55.9%
Research administration	51.7%	Finance	53.1%
Research compliance	47.2%	Recruitment	50.2%
Finance	46.6%	Scientific communications	44.1%
Recruitment	39.5%	Statistical methods	41.3%
Entrepreneurship science	25.4%	Entrepreneurship science	21.6%

Faculty (73.9%; n = 119) and staff (87.3%; n = 165) reported that additional research training/learning opportunities would give them more confidence in completing their job responsibilities. When asked to rank the effectiveness of training methods, 54.9% of faculty and 60.6% of staff considered live courses (in-person) and workshops most effective, whereas 35.2% of faculty and 22.9% of staff ranked online courses and workshops as most effective (Table 3). When asked to rank the likelihood of attending live, blended, or online trainings, 52.9% (n = 64) of faculty and 58.0% (n = 98) of staff indicated they would most likely attend an online training.

**Table 3.** Effectiveness of training methods in meeting research learning goals

Method	Effectiveness	Faculty (122)	Staff (170)	All (292)
Online	Most effective	35.2%	22.9%	28.1%
	Moderately effective	48.4%	61.2%	55.8%
	Least or not effective	16.4%	15.9%	16.1%
Blended (online and live)	Most effective	47.5%	44.1%	45.5%
	Moderately effective	44.3%	47.6%	46.2%
	Least or not effective	8.2%	8.3%	8.2%
Live	Most effective	54.9%	60.6%	58.2%
	Moderately effective	33.6%	30.6%	31.8%
	Least or not effective	11.5%	8.8%	9.9%
Resource library	Most effective	36.9%	23.5%	29.1%
	Moderately effective	41.8%	50.6%	46.9%
	Least or not effective	21.4%	25.9%	14.0%

Out of 290 individuals who responded to the question about whether they knew where to find existing research training opportunities, 38.6% (n = 112) indicated that they did not know and 14.1% (n = 41) reported that they had never looked. A total of 279 respondents provided their preferences for learning about new research training and professional development opportunities. More than three quarters, 77.4% (n = 216) indicated that they preferred emailed newsletters. Websites and recommendations from a supervisor or colleague were preferred by another 9.3% (n = 26) and 6.8% (n = 19) respondents, respectively. Seventy-five percent of faculty and staff considered a centralized online location that provided a comprehensive list of research training and professional development opportunities as very valuable.

### Qualitative Results

The most frequently cited barrier described by both faculty and staff as preventing them from participating in training opportunities was time. Most respondents described concerns with capacity, conflicting obligations, and the lack of time to attend trainings. This was described by one faculty respondent as, “Time to attend. Between clinic and patient care, there’s just never time.” Some respondents suggested recorded sessions, minitrainings in lieu of retreats, or offering sessions at varying times as ways to overcome scheduling conflicts. The next most common barrier to participating in training was a lack of awareness of learning opportunities and resources. This was a more common complaint among staff than faculty, as many staff indicated they were not being notified of trainings and were unsure of where to look for opportunities. As one staff member noted, “Lack of knowledge as to where I would find a list of courses or where I would sign up for them. I’ve only known about trainings that were required at the start of my job or that my supervisor informed me of.” The third most common barrier was indicative of a large diverse organization and was described as a lack of relevant offerings. Responses varied based on the respondent’s field of interest. In some cases, respondents felt the trainings were not relevant because sessions were primarily geared towards basic scientists, while others noted the majority of trainings were targeting clinical researchers. Multiple respondents noted that the trainings were too broad or entry level. In some cases, the respondent was unable to ascertain if the training was relevant. Suggestions were made to include more details on course content or provide the course syllabi to elucidate the training objectives and benefits. Finally, a few respondents indicated the in-person format of the training as a barrier despite the fact that the survey was administered pre-COVID-19. This was frequently cited by staff or faculty who worked remotely or in clinics that were not located within the primary university campus. Virtual or recorded formats were suggested as ways to improve attendance for those in remote locations.

When asked about specific trainings that would be valuable, the common theme was more training on internal and external research processes. Yet, the responses to this question included a wide array of suggestions. Examples ranged from training on changes in the National Institutes of Health submission software system to internal processes spanning the development and implementation of studies such as writing and submitting a grant, IRB requirements, vendor selection, and contracting. As one faculty member noted, “Financial, grants management, and personnel management are all the things I truly need to be successful. The science stuff is sadly the easy part.” On a similar note, a staff member stated, “I spend a lot of time trying to navigate the shifting waters of bureaucracy. It would help to know as much as possible about the infrastructure that we are trying to do research in.” Related to research design and methods, respondents noted interest in statistics, cost-effectiveness, and dissemination and implementation science training. Respondents also noted the importance of additional trainings in mentorship, work-life balance, and communication skills.

## Discussion

Our survey gathered information from our research workforce that included a good representation of faculty and staff in various research-related roles. The majority of respondents work in the COM, which is expected as this is the largest college at our institution with the largest research portfolio. A greater proportion of responses were from women at both the faculty and staff levels. This is consistent with the make-up of clinical and translational research staff at the institution and with attendance at CTSA research trainings. Respondents had a range of experience at our institution (average 5 years) and in research (average 11 years) and represented basic, clinical, and translational research. Our survey was distributed by email, utilizing an all faculty listserv and staff list narrowed by state job classifications. Despite our effort to focus the recipient list to those involved in research, it is likely that many who received the invitation had no research involvement. These individuals may have disregarded the email message rather than opening the survey and opting out. Taking this into account, we consider the overall response rate of nearly 22% to provide a good representation of our clinical and translational research workforce.

The need to train both faculty and staff to meet the evolving needs of academic institutions and colleges is well recognized [9]. Understanding faculty and staff priorities for training are critical for focusing our resources on developing valuable learning opportunities. Faculty and staff both rated grant development, research project development, and professional development among their top five areas viewed as very valuable for further training. Faculty also included statistical methods and dissemination and implementation, while staff included research compliance and research administration in their top five areas. It is plausible that specific training priorities within these broad topic areas vary for faculty and staff because of the differences in roles and responsibilities for research that exist for faculty compared to staff.

The majority of faculty responding to our survey viewed their role in research as that of the lead or contributing investigator and thus have agreed to comply with policies established by agencies that oversee and fund their research. Specifically, the United States Food and Drug Administration and International Council for Harmonization of Technical Requirements for Pharmaceuticals for Human Use provide guidance on the role of investigators and what can be delegated to members of the study team [10,11]. Similarly, sponsors such as NIH have defined investigator roles and responsibilities [12].

Moreover, the duties that fall within the scope of a topic such as research administration vary widely, which could explain the high level of interest across staff respondents from various job classifications and point to the need for comprehensive training. A recent survey of clinical research coordinators showed lower levels of perceived competence in areas related to regulations and product development [13]. The priority placed on trainings in research compliance and research administration may be explained by the majority of responding staff self-identifying as research coordinators. Furthermore, training priorities may vary by career stage and experience level [14]. These most valued training areas will be the priority for our training opportunity development efforts. Results from the survey will be used to direct further inquiry to better understand which aspects of grant, research project, and professional development are most needed for faculty and staff so that future training is targeted for the needed aspects of these topics and at the optimal level (introductory, advanced, etc.).

When asked about preferences for training opportunities, most faculty and staff indicated that live trainings were more effective than online or blended training opportunities or a resource library. However, the majority of faculty and staff indicated that they were more likely to attend online trainings. Time was indicated most often as the barrier to training in open-ended responses and provides further endorsement of the need for designing training with flexibility in the mode of delivery. This is consistent with previous surveys of faculty at our institution who indicated that time was a significant barrier to mentoring and being mentored [3]. Time has also been identified as a barrier to continuing medical education for clinical providers [15]. Together, these results speak to the number of responsibilities placed on the clinical and translational research workforce and the importance of accessibility to training opportunities. Well-intentioned faculty and staff may want to increase their knowledge and skills but may not have the time available to attend a live course. One caveat with interpreting the results of these questions is that we did not specify whether the online training was synchronous (live) or asynchronous. Based on our use of the term live to indicate in-person training and the similarity of the effectiveness results for the online and resource library options, we believe that most respondents interpreted online as asynchronous. It is important to note that this survey was conducted pre-COVID-19 and was thus not influenced by the transition to largely virtual work environments because of the COVID-19 pandemic. We would expect different results in the perception of the effectiveness of online training if we conducted this survey post-COVID-19 and indicated that online training was synchronous. Survey responses were used to guide the transition of training and learning opportunities for our clinical and translation research workforce during the sudden evolution to remote work brought about by the pandemic. This transition included a rapid pivot to online and virtual platforms, options for synchronous and asynchronous participation, and enhanced communication of training and professional development offerings.

Creating training opportunities will not improve our clinical and translational research workforce’s readiness if they are not aware of the offerings. Approximately 50% of respondents either did not know where to look or have never sought research-related training opportunities. At one level, this finding may indicate a need for increased marketing of research training opportunities. It is also plausible that this reflects the need for greater awareness of the importance of professional development for the clinical research workforce. Respondents overwhelmingly indicated that emailed newsletters such as our weekly CTSA newsletter were the preferred method to learn about training opportunities, which will help to focus our marketing efforts. There was widespread support for creating a comprehensive, centralized online list of research training and professional development opportunities available to faculty and staff. The creation of a centralized portal for research training opportunities is an aim of the TWD during our current award period, so we were encouraged that 75% of respondents also viewed this as a need.

We will use the information gained from the survey in four main ways. One, we will review the currently offered trainings that fall under the top five areas of value as identified by faculty and staff to determine if we are missing key topics or if the issue is a lack of knowledge of the available trainings. Two, we will increase our communications related to trainings via email newsletters, as the preferred method indicated by survey respondents. Three, we will identify areas where new trainings are needed. Additional surveys and focus groups with input from research stakeholders representing different research disciplines, career levels, and roles will elucidate specific needs within the top five areas. This will inform our understanding, for example, of what is meant by professional development. Four, we will review the quality of our online trainings to increase the perception of and overall effectiveness for the clinical and translational research workforce. We have several high-quality, online graduate programs at Medical University of South Carolina as well as faculty who have PhDs in education that can serve as resources for improving the effectiveness of our online trainings.

## Conclusions

The results of our survey of the clinical and translational research workforce at a CTSA hub situated at a free-standing academic medical center indicate the need for training opportunities in grant development, research proposal development, professional development, statistical methods, dissemination and implementation, research compliance, and research administration. In this prepandemic survey, faculty and staff indicated that they were more likely to attend trainings online, even though they felt that live trainings were more effective. This is not unexpected as time constraint was the most common barrier to research training identified by both faculty and staff. Awareness of and access to training opportunities also arose as key barriers, with greater than 50% of respondents unaware of current trainings or how to locate them. We will use these results to focus our efforts to centralize and communicate the availability of research training opportunities. Lastly, our survey shows the importance of assessing the needs of both faculty and staff as each group may have unique needs that might not be met otherwise.

## Supplementary material

For supplementary material accompanying this paper visit https://doi.org/10.1017/cts.2021.875. click here to view supplementary material
