# Supplementary material for: Clinical and translational research workforce education survey identifies needs of faculty and staff
Source: J Clin Transl Sci. 2021 Nov 10;6(1):e8. doi: 10.1017/cts.2021.875 (PMC8826003; doi:10.1017/cts.2021.875)
Supplement: Supplementary file 1 [file S205986612100875Xsup001.pdf]

SCTR Translational Workforce Development Research Training Needs Assessment

The South Carolina Clinical & Translational Research (SCTR) Institute has as part of its overarching mission to develop the clinical and translational research workforce. SCTR's Translational Workforce Development program is conducting a voluntary Research Training Needs Assessment to identify:

- the utilization of existing research training opportunities for staff and faculty at MUSC. the research training needs for these groups.
- barriers and facilitators to research training.
- recommendations and best practices for MUSC and other academic medical centers.

Please contact SCTR-workforcedev@musc.edu if you have any questions.

---

1. Are you involved with the conduct, administration, oversight, or support of research at MUSC?
   - ○ Yes
   - ○ No

1a. Are you involved with basic or clinical research? [select all that apply]
Opens if **"Yes"** is selected for question #1.
- ☐ Basic
- ☐ Clinical
- ☐ Other

Please describe "other".
Opens if **"Other"** is selected for question #1a.
____

1b. Would you like to be involved with research at MUSC?
Opens if **"No"** is selected for question #1.
- ○ Yes
- ○ No If **"No"** is selected, survey ENDS.

If you would like to receive information about research training opportunities at MUSC, please contact SCTR-workforcedev@musc.edu.
Opens if **"Yes"** is selected for question #1b.

Would you like to continue with the needs assessment?
Opens if **"Yes"** is selected for question #1b.
- ○ Yes
- ○ No If **"No"** is selected, survey ENDS.

Background Information

2. Please select your role at MUSC.
   - ○ Staff
   - ○ Faculty

2a. Please indicate your primary role/title at MUSC. If your exact role/title is not listed, please select the role/title that most closely describes your role/title.
Opens if **"Staff"** is selected for question #2.
   - ○ Program/Research Assistant/Intern
   - ○ Program/Research Coordinator
   - ○ Program/Project Manager
   - ○ Program/Project Director
   - ○ Regulatory Specialist
   - ○ Research Analyst/Data Manager
   - ○ Research/Grants Administrator
   - ○ Research Investigator
   - ○ Research/Lab Specialist
   - ○ Research Nurse
   - ○ Other

Please specify your role. Opens if **"Other"** is selected for question #2a. ____________________

2b. Please indicate your primary role/title at MUSC. If your exact role/title is not listed, please select the role/title that most closely describes your role/title.
Opens if **"Faculty"** is selected for question #2.
   - ○ Principal Investigator: leading and developing an independent research program
   - ○ Principal Investigator on a career development research award
   - ○ Co-Investigator/Collaborator: contributing to research
   - ○ Educator
   - ○ Clinician
   - ○ Other

Please specify your role. Opens if **"Other"** is selected from question #2b. ____________________

2c. Please provide your rank.
Opens after *role* is indicated in question #2b.
   - ○ Instructor
   - ○ Assistant Professor
   - ○ Associate Professor
   - ○ Professor
   - ○ Other

Please specify your rank. Opens if **"Other"** is selected for question #2c. ____________________

Please list your job title. ____________________

3. How long have you been in this role? Use "0" if less than 1 year. ____________________
4. How long have you been involved in the field of research? Use "0" if less than 1 year. ____________________
5. Please select your MUSC affiliation
   - ○ Academic Affairs Faculty
   - ○ Dental Medicine
   - ○ Graduate Studies
   - ○ Health Professions
   - ○ Hollings Cancer Center
   - ○ Medicine
   - ○ Nursing
   - ○ Pharmacy
   - ○ Other-MUHA
   - ○ Other

Please list your department or center. Opens if **"Medicine"** is selected for question #5. ____________________
Please describe. Opens if **"Other"** is selected for question #5. ____________________

6. To help us understand your research training/learning needs, please rate the perceived value of the following topics.

	Very Valuable	Somewhat Valuable	Not Valuable
Dissemination & Implementation	○	○	○
Entrepreneurship Science	○	○	○
Finance	○	○	○
Grants Development	○	○	○
Professional Development	○	○	○
Recruitment	○	○	○
Research Administration	○	○	○
Research Compliance	○	○	○
Research Project Development	○	○	○
Scientific Communications	○	○	○
Statistical Methods	○	○	○

Please provide specific examples for trainings you would like in those topics ranked as "Very Valuable".

____

Opens if **"Very Valuable"** is selected as a response for ANY option in question #6.

7. Are there any topics not listed above that would be useful research learning/training opportunities? If so, please describe. ________________________________

8. Please rank the perceived value of the following research learning initiatives.

	Very Valuable	Somewhat Valuable	Not Valuable
Centralized online location/portal for research learning/training opportunities	○	○	○
Comprehensive list of available research learning/training opportunities	○	○	○
Comprehensive list of professional development opportunities	○	○	○
Research orientation for new staff	○	○	○
Other	○	○	○

What should an orientation for new research workforce employees include? ____________
Opens if **"Very Valuable"** or **"Somewhat Valuable"** is selected as a response to *Research orientation for new staff* in question #8.

Please describe "other". ____

Opens if **"Very Valuable"** or **"Somewhat Valuable"** is selected as a response to *Other* in question #8.

9. In the previous 2 years, have you taken part in a research learning/training opportunity at MUSC?

○ Yes
○ No

List any research learning/training opportunities that you have taken as part of your role at MUSC. -

____

Opens if **"Yes"** is selected for question #9.

Please list the entity (e.g., program, division, department) that provided these research learning/ training opportunities. ____
Opens if **"Yes"** is selected for question #9.

10. Please describe any barriers that have prevented you from taking part in research training/ learning opportunities. ____

11. Do you feel that additional research training/learning opportunities would give you more confidence in completing your job responsibilities?
    - ○ Yes
    - ○ No

If **"No"** is selected, survey ENDS.

If you would like to receive information on future research learning opportunities, please provide your email address. Opens if **"Yes"** is selected for question #11.

12. Please rank the effectiveness of the following methods of training for meeting your research training/learning goals.

	Most Effective	Moderately Effective	Least Effective	Not at All Effective
Online course/workshop	○	○	○	○
Blended course (part online, part live)	○	○	○	○
Live course/workshop	○	○	○	○
Resource library	○	○	○	○

13. Please rank the likelihood that you would attend the following methods of training.

	Most Likely	Somewhat Likely	Least Likely
Online course/workshop	○	○	○
Blended learning course (par online, part live)	○	○	○
Live course/workshop	○	○	○

14. I know where to find existing research training/learning opportunities
    - ○ Agree
    - ○ Disagree
    - ○ I have never looked for research training/learning opportunities

15. What is your preferred method for hearing about NEW training/professional development learning opportunities?
    - ○ Ads in public places research
    - ○ Catalyst
    - ○ Email newsletter
    - ○ Social media
    - ○ Supervisor/colleague
    - ○ Website
    - ○ Word of mouth
    - ○ Other

Please describe "other".
Opens if **"Other"** is selected for question #15. ______________________________

16. Please provide any other comments about research training/learning opportunities at MUSC. ______________________________

We are committed to providing resources to a diverse community of clinical and translational research stakeholders. The responses you provide to the questions below will be used for ongoing program evaluation and development. You may choose to not respond.

17. With which group(s) do you identify? Select all that apply.
    - American Indian or Alaskan Native
    - Asian
    - Black or African American
    - Hispanic, Latino, or Spanish origin
    - Middle Eastern or North African
    - Native Hawaiian or Other Pacific Islander
    - White
    - A race, ethnicity, or origin not listed above
    - I prefer not to answer

Please specify how you describe your race, ethnicity, or origin.___________________________________
Opens if **"A race, ethnicity, or origin not listed above"** is selected for question #17.

18. How do you describe your gender identity?
    - Female
    - Male
    - Gender not specified above
    - I prefer not to answer

Please specify how you describe your gender identity.
Opens if **"Gender not specified above"** is selected for question #18.__________________________
